# Peste des petits ruminants virus non-structural C protein inhibits the induction of interferon-β by potentially interacting with MAVS and RIG-I

**DOI:** 10.1007/s11262-020-01811-y

**Published:** 2021-01-03

**Authors:** Li Linjie, Shi Xiaoling, Ma Xiaoxia, Cao Xin, Amjad Ali, Bai Jialin

**Affiliations:** 1 Key Laboratory of Bioengineering & Biotechnology of the National Ethnic Affairs Commission, Biomedical Research Center, Northwest Minzu University, Lanzhou, 730030 China; 2 School of Chemical and Biological Engineering, Lanzhou Jiaotong University, Lanzhou, 730070 China; 3 College of Life Science and Engineering, Northwest Minzu University, Lanzhou, 730030 China

**Keywords:** Protein C, Interferon-β (IFN-β), Luciferase reporter assay, Pest des petits ruminants virus, Antiviral state

## Abstract

Peste des petits ruminants virus (PPRV) causes an acute and highly contagious disease in domestic and wild small ruminants throughout the world, mainly by invoking immunosuppression in its natural hosts. It has been suggested that the non-structural C protein of PPRV helps in evading host responses but the molecular mechanisms by which it antagonizes the host responses have not been fully characterized. Here, we report the antagonistic effect of PPRV C protein on the expression of interferon-β (IFN-β) through both MAVS and RIG-I mediated pathways in vitro. Dual luciferase reporter assay and direct expression of IFN-β mRNA analysis indicated that PPRV C significantly down regulates IFN-β via its potential interaction with MAVS and RIG-I signaling molecules. Results further indicated that PPRV C protein significantly suppresses endogenous and exogenous IFN-β-induced anti-viral effects in PPRV, EMCV and SVS infections in vitro. Moreover, PPRV C protein not only down regulates IFN-β but also the downstream cytokines of interferon stimulated genes *56 (ISG56)*, *ISG15*, C-X-C motif chemokine (*CXCL10*) and RIG-I mediated activation of IFN promoter elements of ISRE and NF-κB. Further, this study deciphers that PPRV C protein could significantly inhibit the phosphorylation of STAT1 and interferes with the signal transmission in JAK-STAT signaling pathway. Collectively, this study indicates that PPRV C protein is important for innate immune evasion and disease progression.

## Introduction

Peste des petits ruminants (PPR) is an acute and highly contagious disease of both domestic and wild small ruminants, caused by peste des petits ruminants virus (PPRV), a Morbillivirus of the *Paramyxoviridae* family [1]. PPR is causing serious economic losses in developing countries where sheep and goats rearing/business make an important component of the livelihood of the poor [2]. Other important members within the *Paramyxoviridae* family are measles virus (MeV), phocine distemper virus (PDV), rinderpest virus (RPV), cetacean morbillivirus (CeMV), feline morbillivirus (FMV) and canine distemper virus (CDV) [3]. PPRV is a single-stranded, negative-sense, non-segmented RNAvirus with a genome size of up to 15 Kb, having six genes, encoding eight structural and non-structural proteins. Structural proteins are; the fusion protein (F), hemagglutinin membrane glycoprotein (H), large protein (L), matrix protein (M), phosphoprotein (P), and nucleoprotein (N), whereas W and C/V proteins are the non-structural proteins [4, 5]. Co-transcripts of the *P* gene as a result of G insertion; single and/or double, at the editing site during transcription, leads to V and W proteins synthesis. Likewise, C protein coding mRNA is also generated from the second ATG codon of the alternate open reading frame (ORF) of the P cistron [6–8]. During the course of infection and for minimizing host’s interferon (IFN) responses, both C and V proteins are considered to be important [9–12].

Viruses are able to use various strategies to evade the immune responses including the induction of immunosuppression by offsetting host IFN responses, stimulated via pathogen-associated molecular patterns (PAMPs) by cellular pathogen recognition receptors (PRRs). Retinoic acid-inducible gene I (RIG-I)-like receptors (RLR), a PRR, which include important proteins like melanoma differentiation-associated protein 5 (MDA-5), RIG-I, and laboratory of genetics and physiology 2 (LGP2), can detect intracellular pathogens by recognizing specific pathogen-associated RNAs. These receptors contain a DExH/D box RNA helicase domain and a specific carboxy-terminal domain, important for host immunity. Moreover, the two tandem caspase recruitment domains (CARDs) at the amino-termini of MDA-5 and RIG-I are presumed to have a role in the downstream signaling which activates IFN-β promoter [13]. LGP2, lacking CARD, on the other hand, activates and represses MDA-5 and RIG-1, respectively [14–16]. LGP2 role in PAMP recognition by MDA-5 and RIG-I has also been suggested [17].

Paramyxovirus V protein on binding to MDA-5, blocks MDA-5-mediated IFN-β induction [18–20]. Dephosphorylation of MDA-5, necessary for MDA-5 downstream signaling applications, has been shown to be blocked by MeV V protein when binds to phosphoprotein phosphatase 1 (PP1) [21]. Likewise, the V protein of parainfluenza virus type 5 (PIV5) does not interact with RIG-1 [19], but binds to LGP2 [22, 23], thereby inhibits IFN-β activation by RIG-1 pathway [22]. Moreover, studies indicate that IFN-β promoter activation by MDA-5 can be facilitated by LGP2 whereas PIV5 V may block MDA-5 signals by interacting with LGP2 [22, 24]. There have been three studies reporting that PPRV V protein blocks IFN-β induction by binding to MDA-5 [12, 20, 25]. Hence, it is evident that paramyxovirus V protein plays an important role in the inhibition of IFN-β induction by interfering with MDA-5 and RIG-I pathways.

The exact mechanism by which paramyxovirus C proteins interact with IFN-β is not clear. C protein of morbillivirus has been shown to play a role in the regulation of viral RNA synthesis, replication, and translation which could lead to suppression of IFN-β promoter [26–29]. Engineered viruses, incapable of producing C proteins, exhibit PAMP in the form of generating more double-stranded RNA (dsRNA) which ultimately activates IFN-β promoter via protein kinase R (PKR) [30, 31] and MDA-5 during infection, unlikely wild type viruses [32–34]. It has also been proposed that C protein can block IFN-β transcription directly [35] and that IFN-β induction by MDA-5 and RIG-I mediated pathways has not been affected by PPRV, lacking C gene/protein, albeit they did not find any direct evidence that it blocks either MDA-5 or RIG-I pathways [12]. Therefore, in order to have a deep understanding of the molecular mechanism, we investigated the effect of PPRV C protein on RIG-1 and MAVS mediated induction of IFN-β, and its downstream activation of other cytokines and its role in PPRV infection.

## Materials and methods

### Cells, viruses, antibodies, and reagents

Human embryonic kidney 293T cells (HEK-293T) and African Green Monkey Kidney Cells (Vero) were grown in DMEM medium (Life Technologies, CA, USA), supplemented with heat-inactivated 10% fetal bovine serum (FBS; Lanzhou Minhai Biotechnology Co. Ltd, Lanzhou, China), 100 U/ml penicillin and 100 µg/ml streptomycin (Life Technologies, CA, USA) in a 5% CO_2_ incubator at 37 ˚C. Nigeria 75/1 attenuated vaccine strain of PPRV, purchased from Xinjiang TianKang Animal Husbandry Biotechnology Co. Ltd, was amplified and used to inoculate Vero cells with. Vesicular stomatitis virus (VSV, ATCC VR-1415) and encephalomyocarditis virus (EMCV, GS-01 strain; KJ524643) available at Key Laboratory of Bioengineering and Biotechnology of the National Ethnic Affairs Commission, China, were used in subsequent experiments.

Viral titrations as 50% tissue culture infectious dose (TCID_50_) from infected vero cells were calculated using Reed-Muench method. Mouse anti-Flag monoclonal antibody (mAb), rabbit anti-HA, anti-β-Actin and anti-phospho-STAT1 (Tyr^701^) mAbs, mouse anti-STAT1 mAbs and horse radish peroxidase (HRP)-conjugated sheep anti-mouse IgG and anti-rabbit IgG were purchased from Cell Signaling Technology (USA). TRIzol™ Reagent (Ambion), Opti-MEM (Gibco), and Lipofectamine 2000 (Invitrogen) were purchased from Thermo Fisher Scientific. Dual Luciferase Reporter Gene Assay Kit was purchased from Beyotime Biotechnology (Shanghai, China). Protease and phosphatase inhibitor cocktails were purchased from Roche Pharmaceutical Ltd, Switzerland. ECL substrate luminescence kit (Millipore), PVDF membranes and IFN-β were purchased from Sigma-Aldrich, St. Louis, MO, USA.

### Plasmids and transfection assays

pCMV-HA and pMD18-T plasmids were purchased from TaKaRa Medical Biotechnology (Beijing) Co. LTD. pGL3-IFN-β, pGL3-ISRE, pGL3-NF-κB, pRL-TK, pRK-Flag-RIG-IN, pRK-Flag-MAVS, pRK-Flag-MAD5, pRK-Flag-TRAF3, pRK-Flag-TBKI, pRK-Flag-IKKε, pRK-Flag-IRF3, and pRK-Flag-IRF7 were kindly provided by Prof. Qiyun Zhu from Lanzhou Veterinary Research Institute, Chinese Academy of Agricultural Sciences, while expression plasmid, pCMV-HA-C, was constructed in-house.

HEK-293T cells (2 × 10^5^) in 6-well plates were cultured (70–80% confluency) in DMEM and were transfected with pCMV-HA (2.5 µg), pCMV-HA-C (2.5 µg) and pRK-Flag-RIG-IN (1 µg) alone, and/or pCMV-HA-C (1.5 µg) and pRK-Flag-RIG-IN (1 µg) together using Lipofectamine 2000 Transfection Reagent according to the manufacturer’s instructions. pCMV-HA was added to ensure cells received equal amount of plasmids in each transfection. At 24 h post transfection (hpt), cells were harvested for subsequent analysis.

In order to determine the potential interaction between PPRV C protein and endogenous IFNs, the supernatants of respective cultures were harvested at 24 hpt and I ml of the supernatant was mixed with 1 ml of fresh DMEM medium and were added to fresh HEK-293 cultures and incubated for 24 h, followed by infection with PPRV at 1 multiplicity of infection (MOI), VSV at 0.1 MOI and EMCV at 0.1 MOI.

Similarly, for assessing the interaction between PPRV C protein and exogenous IFNs, the HEK-293 cells (2 × 10^5^), cultured in 6-well plates were transfected with pCMV-HA-C and pCMV-HA for 24 h and were treated with IFN-β (1000 U/ml) for another 24 h, followed by infection with PPRV, VSV and EMCV as discussed earlier. At 24 h post infection (hpi), cells were harvested for RT-qPCR and western blot analysis.

### Quantitative real-time PCR (qPCR)

Total RNA was extracted from cultures by TRIzol™ Reagent according to the manufacturer’s instructions. First strand cDNA was generated using GoScript™ Reverse Transcriptase System (Promega) according to the manufacturer’s instructions. qPCR was performed with SYBR Green PCR Master Mix (Bio-Rad) on a CFX Connect Real-Time System (Bio-Rad) for the expression of *IFN-β*, C-X-C motif chemokine ligand 10 (*CXCL-10*), interferon-stimulated genes 56 (*ISG56*) and *ISG15* and for *L* of VSV, *VP1* of EMCV, and *H* of PPRV. Values obtained for each gene were normalized to that of the gene encoding glyceraldehyde-phosphate dehydrogenase (*GAPDH*). Gene specific primers used in this study are listed in Table 1.

**Table 1:** Gene-specific primers used in the study

Primer name	Primer sequence (5′–3′)
IFN-β-F	5′-GCTTGGATTCCTACAAAGAAGCA-3′
IFN-β-F	5′-ATAGATGGTCAATGCGGCGTC-3′
ISG56-F	5′-TCATCAGGTCAAGGATAGTC-3′
ISG56-R	5′-CACACTGTATTTGGTGTCTAGG-3′
ISG15-F	5′-AGGACAGGGTCCCCCTTGCC-3′
ISG15-R	5′-CCTCCAGCCCGCTCACTTGC-3′
CXCL10-F	5′-GTGGCATTCAAGGAGTACCTC-3′
CXCL10-R	5′-TGATGGCCTTCGATTCTGGATT-3′
H of PPRV-F	5′-CTGAATACCAACATTGAG-3′
H of PPRV-R	5′-GAGGAACTTAATCTTATCG-3′
VP1 of EMCV-F	5′-GCTGTCTGGTATAATGGA-3′
VP1 of EMCV-R	5′-TGATACAGTACAATTSTTTTGGGAC-3′
L of VSV-F	5′-GAGACTTTCTGTTACGGGATCTGG-3′
L of VSV-R	5′-TTGCGGCCGCATGTCAACAAGGGGCTGGAATGTAT-3′
GAPDH-F	5′-CACAAGCTTCCCGTTCTCAG-3′
GAPDH-R	5′-GAGTCAACGGATTTGGTCGT-3′

### Dual luciferase reporter assay

HEK-293T cells (1 × 10^5^) in 12-well plates were cultured in DMEM to 70 ~ 80% confluency and were transfected with internal plasmid, pRL-TK (10 ng) and reporter plasmids, pGL3-IFN-β (100 ng), pGL3-ISRE (100 ng), and pGL3-NF-κB (100 ng) in each well. Then cells were transfected with pCMV-HA (500 ng) and pRK-Flag-RIG-IN (100 ng) alone, and with pCMV-HA-C (400 ng) and pRK-Flag-RIG-IN (100 ng) together using Lipofectamine 2000 Transfection Reagent according to the manufacturer’s instructions. Empty vectors were added to ensure cells received equal amount of plasmids in each transfection. At 24 hpt, cell lysates were prepared and analyzed for Firefly and Renilla luciferase activity using a dual-luciferase reporter assay system (GLoMax 20/20, Promega, USA), according to the manufacturer’s protocol.

For screening other components of the RIG-I mediated pathway, HEK-293T cells in 12-well plates were transfected with internal plasmid, pRL-TK (10 ng) and reporter gene plasmids in each well. Then cells were transfected with pGL3-IFN-β (100 ng) and pRK-Flag-RIG-IN (100 ng), pRK-Flag-MAVS (100 ng), pRK-Flag-IKKε (100 ng), pRK-Flag-TBKI (100 ng), pRK-Flag-TRAF3 (100 ng), pRK-Flag-IRF3 (100 ng), pRK-Flag-IRF7 (100 ng) alone and/or together with pCMV-HA-C (400 ng) in each well. Empty vectors were added to ensure cells received equal amount of plasmids in each transfection. At 24 hpt, cell lysates were prepared and analyzed for Firefly and Renilla luciferase activity using a dual-luciferase reporter assay kit.

### Western blotting

Cells were washed with phosphate-buffered saline (PBS) and lysed using NP40 cell lysis buffer (20 mM Tris–HCl, 150 mM NaCl, 1 mM EDTA, 1% Nonidet P-40) supplemented with a protease and phosphatase inhibitor cocktails for 30 min at 4˚C. Cells lysates were subjected to 10% SDS-PAGE electrophoresis and were subsequently transferred to a PVDF membrane. Membranes were blocked by 5% skimmed milk for 1 h, followed by incubation with appropriate antibodies at 4˚C overnight. The membranes were exposed to chemiluminescence reagents as described previously [36]. Mouse anti-Flag mAb, rabbit anti-HA mAb, rabbit anti-phospho-STAT1 (Tyr^701^) mAb and mouse anti-STAT1 mAb were used to detect the expression of RIG-IN, HA, total STAT1, and phosphorylated STAT1, respectively. β-actin was used as a loading control.

### Statistical analysis

Statistical analysis was performed using GraphPad Prism version 5.0 (GraphPad Software, San Diego, CA, USA). Data is presented as mean ± SEM with an error bar that represents at least three independent experiments. Differences in indicators between treatment groups and controls were assessed using Student’s *t*-test. A two-tailed test with *p* value < 0.05 corresponds to statistically significant difference and is marked as “*” while *p* < 0.01 and *p* < 0.001 are marked as “**” and “***” respectively, indicating higher significant differences.

## Results

### PPRV C protein antagonizes INF-β and its downstream cytokines induced by RIG-I in RLR signaling pathway

**Fig. 1:**
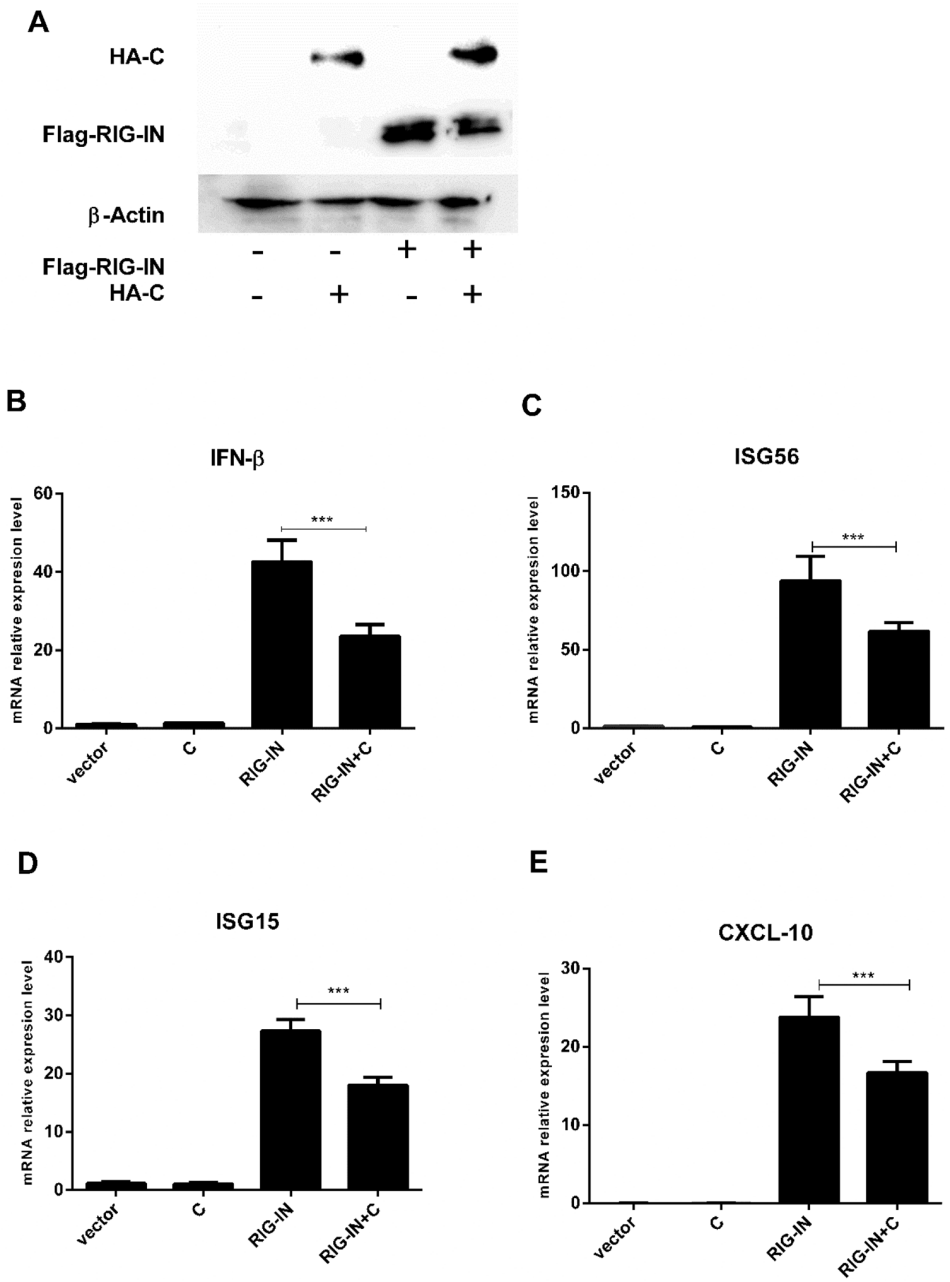
PPRV C protein inhibits the gene expression of *INF-β* and its downstream factors induced by RIG-IN at transcriptional level. HEK-293T cells (2 × 10^5^) in 6-well plates were cultured (70–80% confluency) and were transfected with pCMV-HA (2.5 µg), pCMV-HA-C (2.5 µg), pRK-Flag-RIG-IN (1 µg) alone, and co-transfected with pCMV-HA-C (1.5 µg) and pRK-Flag-RIG-IN (1 µg) using Lipofectamine 2000 Transfection Reagent. At 24 h post transfection (hpt), the expression of Flag-RIG-IN and HA-C was detected by western blotting with anti-Flag mAb and anti-HA mAb, respectively. The expression of *β-actin* was used as a loading control. The mRNA expression of each *IFN-β*, *ISG56*, *ISG15* and *CXCL-10* was measured by RT-qPCR. **a** Western blotting has shown that HA-C and Flag-RIG-IN expressed in the transfected cells. **b** The mRNA expression of *IFN-β* has been reduced to almost half under the effect of PPRV C as compared to RIG-1N. Similarly, mRNA expression of *ISG56* (**c**) *ISG15* (**d**) and *CXCL-10* (**e**) have been down-regulated by *PPRV C* expression

Initially, western blot (WB) was performed to determine the expressions of PPRV C and RIG-I proteins when pCMV-HA-C and pRK-Flag-RIG-IN were co-transfected into HEK-293T cells (Fig. 1a). The qPCR analysis indicated that PPRV C significantly (*p* < 0.001) down-regulated *IFN-β*, *ISG56*, *ISG15* and *CXCL10*, induced by RIG-IN (Fig. 1b to e).

### PPRV C protein hampers type I interferon response in vitro

**Fig. 2:**
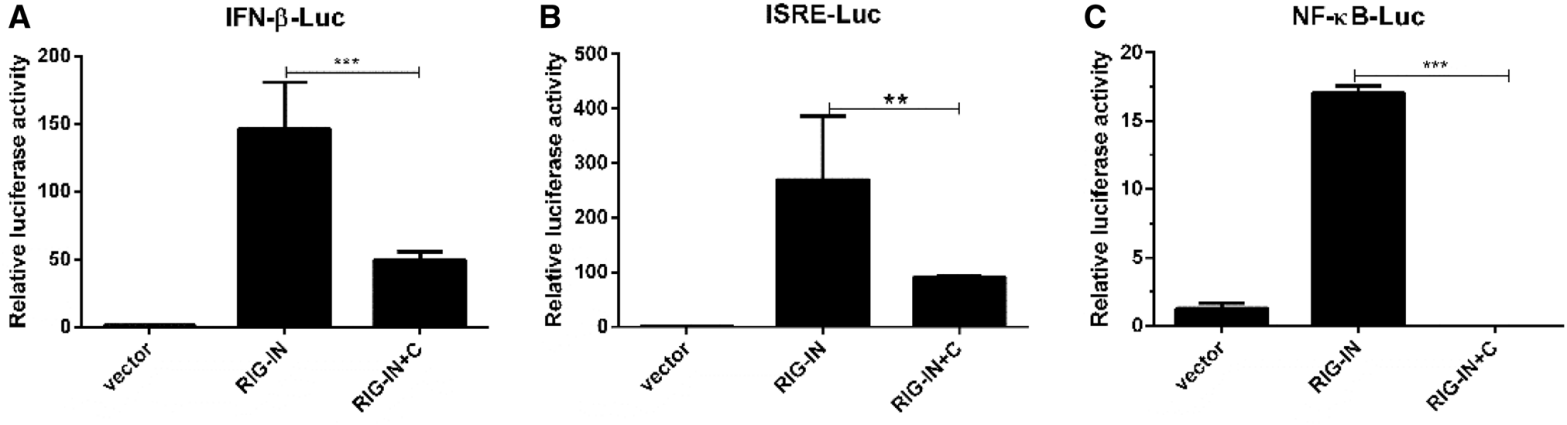
PPRV C protein inhibits RIG-IN mediated reporter gene activity. HEK-293T cells (1 × 10^5^) in 12-well plates were cultured (70–80% confluency) and were transfected with internal plasmid pRL-TK (10 ng) along with reporter plasmids pGL3-IFN-β (100 ng) (**a**), pGL3-ISRE (100 ng) (**b**) and pGL3-NF-κB (100 ng) (**c**) in each well, respectively. Then cells were transfected with pCMV-HA (500 ng), pCMV-HA-C (400 ng) and pRK-Flag-RIG-IN (100 ng) alone, and with pCMV-HA-C (400 ng) and pRK-Flag-RIG-IN (100 ng) together using Lipofectamine 2000 Transfection Reagent. Empty vectors were added to ensure cells received equal amount of plasmids in each transfection. At 24 hpt, cell lysates were analyzed for Firefly and Renilla luciferase activity using a dual-luciferase reporter assay kits. The expression levels of *IFN-β* (**a**), *ISRE* (**b**) and *NF-κB* (**c**) were significantly reduced in pCMV-HA-C and pRK-Flag-RIG-IN co-transfected cells than that of pRK-Flag-RIG-IN transfected cells

Dual luciferase reporter assay was performed to detect whether PPRV C protein affects the activities of IFN-β and its downstream IFN stimulated response element (ISRE) and nuclear factor kappa-B (NF-κB). The relative luciferase activity of reporter genes of *IFN-β*, *ISRE* and *NF-κB* observed in co-transfected pRK-Flag-RIG-IN and pCMV-HA-C cells was significantly less than in transfected pRK-Flag-RIG-IN cells as indicated in Fig. 2; panels a (*p* < 0.001), b (*p* < 0.01), and c (*p* < 0.001), respectively. This indicates that C protein of PPRV checks RIG-I mediated IFN-β at transcriptional level and antagonizes antiviral response.

### Screening of the target molecules in RLR signaling pathway

**Fig. 3:**
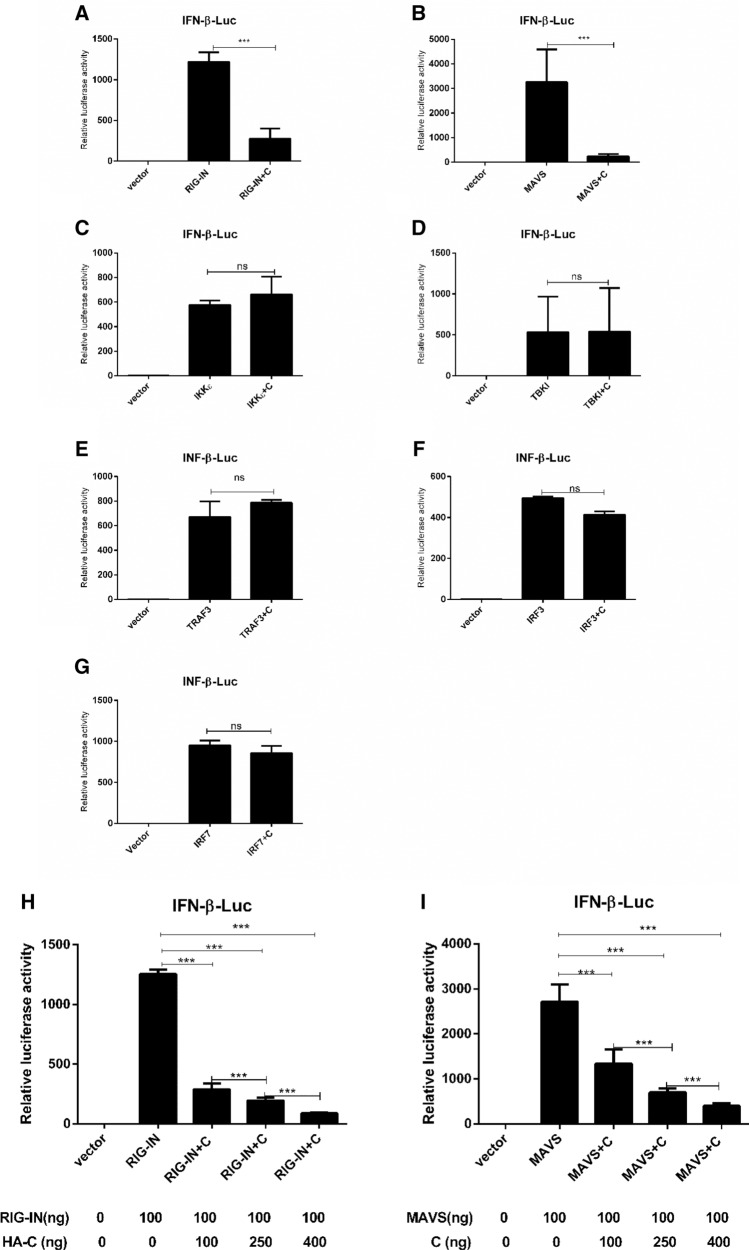
Effect of the expression of the seven possible target molecules of PPRV C protein-induced activation of *IFN-β* promoter in RLR pathway. Monolayer HEK-293T cells in 12-well plates were transfected with internal reference gene plasmid pRL-TK (10 ng) and reporter gene plasmid pGL3-IFN-β (100 ng) in each well. Cells were then transfected with pRK-Flag-RIG-IN (100 ng), pRK-Flag-MAVS (100 ng), pRK-Flag-IKKε (100 ng), pRK-Flag-TBKI (100 ng), pRK-Flag-TRAF3 (100 ng), pRK-Flag-IRF3 (100 ng), and pRK-Flag-IRF7 (100 ng) alone and together with pCMV-HA-C (400 ng) in each well. For in depth analysis, various doses of pCMV-HA-C (100, 250 or 400 ng) against constant values of either pRK-Flag-RIG-IN (100 ng) or pRK-Flag-MAVS (100 ng) were used (**h** and **i**). Empty vectors were added to ensure cells received equal amount of plasmids in each transfection. At 24 hpt, cell lysates were analyzed for Firefly and Renilla luciferase activity using a dual-luciferase reporter assay kit. Expression of *IFN-β* responsive promoter under the effect of C protein was significantly lower than activated by RIG-I (**a**) and by MAVS (**b**). On the contrary, the effect of C protein on the expression levels of *IKKε* (**c**), *TBKI* (**d**), *TRAF3* (**e**), *IRF3* (**f**), and *IRF7* (**g**) was not significant. **h** RIG-I-mediated *IFN-β *and MAVS-mediated *IFN-β* promoter activation were found to be significantly inhibited by C protein (**h** and **i** respectively) and were dose dependent; increase in dose from 100 to 400 ng halted IFN-β expression in each case

To evaluate whether PPRV C protein interacts with RIG-I, mitochondrial antiviral signaling (MAVS), I-kappa kinase (IKKε), TRAF family member-associated NF-κB activator (TANK)-binding kinase 1 (TBKI), tumor necrosis factor receptor associated factor 3 (TRAF3), interferon regulatory factor 3 (IRF3), and IRF7, dual luciferase reporter gene assays were carried out using RIG-I responsive reporters pRK-Flag-RIG-IN, pRK-Flag-MAVS, pRK-Flag-IKKε, pRK-Flag-TBKI, pRK-Flag-TRAF3, pRK-Flag-IRF3, and pRK-Flag-IRF7, respectively (Fig. 3). Expression of *IFN-β* responsive promoter under the influence of C protein was significantly lower than activated by RIG-IN (Fig. 3a, *p* < 0.001) and by MAVS (Fig. 3b, *p* < 0.001). On the other hand, the effect of the C protein on the expressions of *IKKε*, *TBKI*, *TRAF3*, *IRF3,* and *IRF7* was not significant (*p* > 0.05) (Fig. 3c, d, e, f, and g).

In order to evaluate whether IFN-β suppression by C protein is dose-dependent, HEK-293T cells were transfected with gradient doses of pCMV-HA-C (100, 250 or 400 ng) against a constant concentration of pRK-Flag-RIG-IN (100 ng) or pRK-Flag-MAVS (100 ng) for 24 h and were analyzed. Analysis indicated that with the increase in concentration of pCMV-HA-C from 100 to 400 ng, the expression of RIG-I mediated *IFN-β* diminished significantly (Fig. 3h). Similarly, results demonstrated that C protein significantly (*p* < 0.001) inhibits MAVS-mediated *IFN-β* promoter activation in a dose dependent manner as shown in Fig. 3i.

### PPRV C protein promotes virus replication via decreasing the endogenous and exogenous IFN responses

**Fig. 4:**
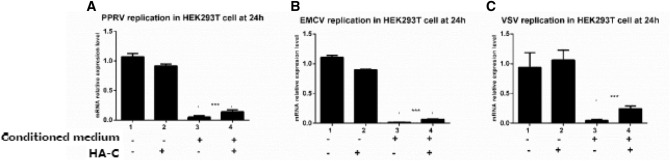
PPRV C protein inhibits the anti-viral effect via decreasing the production of endogenous IFN response. HEK-293T cells (2 × 10^5^) in 6-well culture plates were cultured in DMEM supplemented with 10% FBS for growing to 70–80% confluency. Cells were transfected with pCMV-HA (2.5 µg), pCMV-HA-C (2.5 µg) and pRK-Flag-RIG-IN (1 µg) alone, and pCMV-HA-C (1.5 µg) and pRK-Flag-RIG-IN (1 µg) together using Lipofectamine 2000 Transfection Reagent according to the manufacturer’s instructions. Empty vectors were added to ensure cells received equal amount of plasmids in each transfection. At 24 hpt, the supernatants were harvested and then 1 ml supernatants and 1 ml fresh DMEM medium were added to the new HEK-293T cells and cultured for 24 h, followed by infection with 1 MOI PPRV, 0.1 MOI VSV and 0.1 MOI EMCV, respectively. At 24 h post infection (hpi), all infected cells were harvested for RT-qPCR to measure the gene expression of *PPRV H*, *EMCV VP1* and *VSV L*. **a** The expression of *PPRV H* gene in RIG-IN and PPRV C co-transfected cells was most significantly higher than that in RIG-IN transfected cells (*p* < 0.001); **b** The expression of *EMCV VP1* gene in RIG-IN and PPRV C co-transfected cells was most significantly higher than that in RIG-IN transfected cells (*p* < 0.001); **c** The expression of *VSV L* gene in RIG-IN and PPRV C co-transfected cells was most significantly higher than that in RIG-IN transfected cells (*p* < 0.001)

**Fig. 5:**
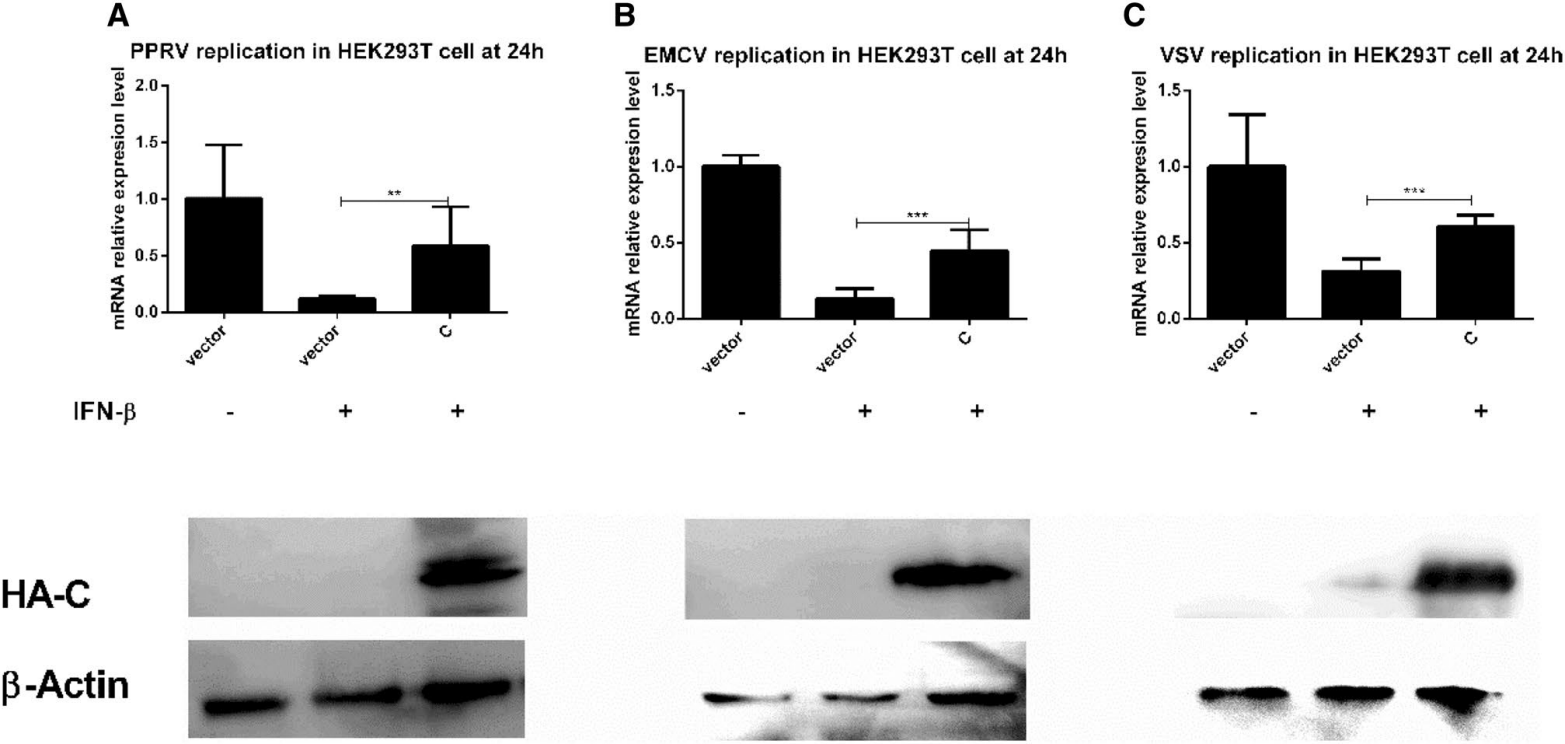
PPRV C protein inhibits the anti-viral effect via decreasing the production of exogenous IFN response. Monolayer HEK-293T cells in 6 well culture plates were transfected with pCMV-HA (2.5 µg) or pCMV-HA-C (2.5 µg). At 24 hpt, these cells were treated with IFN-β (1000 U/ml) for 24 h and then followed by infection with PPRV (1 MOI), VSV (0.1 MOI) and EMCV (0.1 MOI). At 24 hpi, all infected cells were harvested for RT-qPCR to measure the gene expression of *PPRV H*, *EMCV VP1,* and *VSV L*. **a** The expression of *PPRV H* in the transfected HA-C cells treated with IFN-β was significantly higher than that in transfected HA cells (*p* < 0.01); **b** The expression of *EMCV VP1* in the transfected HA-C cells treated with IFN-β was significantly higher than that in transfected HA cells (*p* < 0.001); **c** The expression of *VSV L* in the transfected HA-C cells treated with IFN-β was significantly higher than that in transfected HA cells (*p* < 0.001)

As the expression of RIG-IN can in turn induce the expression of IFN-β, therefore, the supernatant of the over-expressed RIG-IN cells was taken as the source of endogenous IFNs, and will be referred to as conditioned medium. The expression of *H* gene of PPRV in the co-transfected RIG-IN and HA-C was significantly higher (*p* < 0.001) than that in RIG-IN cells at 24 hpi (Fig. 4a). The expression profiles of *VP1* gene of EMCV and *L* of VSV in co-transfected cells at 24 hpi paralleled that of the *H* expression of PPRV (Fig. 4b and c). Moreover, the expression of *H *gene of PPRV in the transfected HA-C cells treated with IFN-β (exogenous) was significantly higher (*p* < 0.01) than that in transfected HA cells at 24 hpi (Fig. 5a). Similarly, the expression of both *VP1* and *L* genes of EMCV and VSV, respectively in HA-C transfected cells treated with IFN-β were significantly higher (*p* < 0.001) than that in HA transfected cells at 24 hpi (Fig. 5b and c). C protein expression as evident by western blot analysis has also been shown in Fig. 5.

### PPRV C protein inhibits the phosphorylation of STAT1

**Fig. 6:**
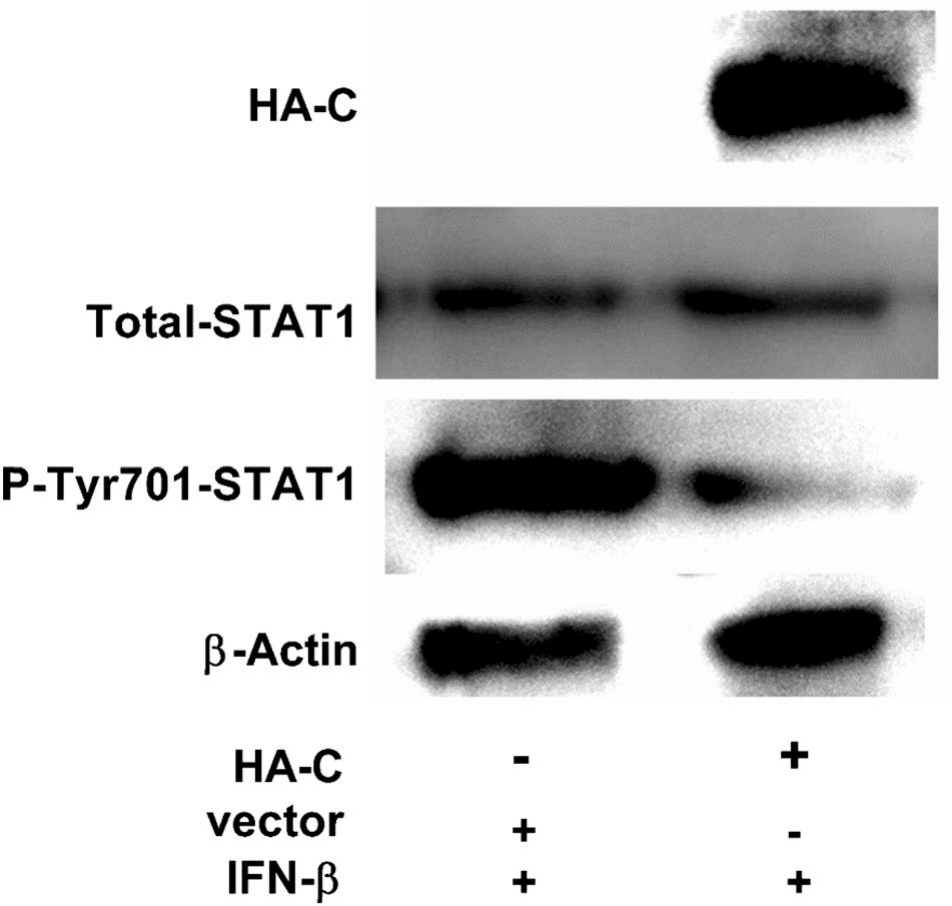
PPRV C protein inhibits the phosphorylation of STAT1. Monolayer HEK-293T cells in 6-well culture plates were transfected with pCMV-HA (2.5 µg) and pCMV-HA-C (2.5 µg), respectively. At 24 hpt, these cells were treated with IFN-β (1000 U/ml). At 24 h post treatment, cells were processed for the expression of total STAT1 and phosphorylated STAT1 with mouse anti-STAT1 mAb and rabbit anti-phospho-STAT1 (Tyr^701^) mAb, respectively. Phosphorylated STAT1 expression was inhibited when C protein was expressed, with no effect on total STAT1. β-actin was used as a loading control. A representative image of the three independent experiments is shown here

To investigate the potential role of PPRV C protein in causing immunosuppression of the host via JAK-STAT pathway, HEK-293T cells were transfected with pCMV-HA-C and pCMV-HA separately and together, and then treated with INF-β for 24 h. Results indicated that the expression of total STAT1 in transfected cells remained unchanged, irrespective of the presence or absence of PPRV C protein (Fig. 6) but the phosphorylation of STAT1 was enhanced when PPRV C was not expressed and decreased when PPRV C was expressed after IFN-β treatment (Fig. 6).

## Discussion

PPRV is one of the major problems of the sheep and goat industries worldwide that inflicts severe economic losses due to high morbidity and mortality rates. Genome of the PPRV expresses C protein, which is a non-structural protein of an alternate open reading frame of *P* cistron and has been thought to be important for bringing about infections [8, 37]. Studies had revealed that C protein though not phosphorylates but binds to the L protein and localizes in the nucleus and cytoplasm [38], thereby contribute to immune evasion and pathogenicity [39–41]. For C protein, in case of Morbillivirus, two mechanisms have been recognized which are responsible for halting IFNs production; by restricting viral replication, thereby decreasing the amount of motifs that PRRs recognizes [28, 29, 42] and direct interference in the PRRs signaling that leads to the inhibition of IFNs transcription [35, 43, 44]. It has been reported that MeV-C and RPV-C proteins of the Morbilliviruses interact with IRF3 in the nucleus and thereby block the activation of IFN-I, though the exact mechanism is not fully known [35, 43]. IRF3-dependent transcription is disrupted by C protein and does not interact with the phosphorylation of IRF3, its dimerization or accumulation [35], indicating that its underlying mechanism is dependent on steric hindrance of IRF3 dimers with the IFN-β promoter. Moreover, it has been observed that MeV-C and V proteins can also block NF-kB pathway, highlighting its vast role in immune suppression and disease progression [44]. Studies also indicate that though V protein is the principal IFNs signaling antagonist during infection [29], MeV-C can also block it [45]. It has also been reported that PPRV lacking C protein did not hamper host IFN-β response [12] but its direct effect was not elucidated.

The exact mechanism by which PPRV evades the immune responses and initiates infection in relation to the C protein is poorly understood. Therefore, we carried out this study in detail to unveil the potential role of PPRV C protein in antagonizing host responses and causing infection by employing HEK-293T cells which is a commonly used, amenable and efficient model system for infection and protein expression studies [46, 47]. Nevertheless, it is a human cell line and so it should be pointed out that it may not be an ideal model for the study of a small ruminant infecting viruses like PPRV.

Here, we report that PPRV C protein significantly antagonizes the expression of *IFN-β* (Fig. 1b) and also interferes with RIG-IN mediated expression of *ISG56*, *ISG15* and *CXCL-10* in vitro as shown in Fig. 1, panels C, D and E, respectively. Dual luciferase assay indicated that C protein of PPRV significantly suppresses *IFN-β*, *ISRE* and *NF-κB* at the transcriptional level (Fig. 2).

Analysis of the target molecules in the RLR pathway indicated that C protein potentially interacts with RIG-I and MAVS (Fig. 3a and b) as compared to the rest of the molecules (Fig. 3c to g) and downregulates their expression substantially that in turn check the IFN-β response. To peruse this interaction further, gradient expression of the C protein was employed. The analysis indicated that a higher expression of C significantly halted the expression of RIG-IN and MAVS-mediated *IFN-β* in a dose dependent manner (Fig. 3h and i). This indicates that C protein plays an important role in counteracting the innate responses which leads to disease progression.

This study further communicates that C protein promotes the replication of PPRV, EMCV and VSV significantly in vitro by halting endogenous IFNs (Fig. 4a, b and c) which implies its importance and a universal role in infection and disease progression. IFN-β as a source of exogenous IFNs was supplied to respective cultures in order to gauge the effect of the C protein with. Analysis revealed that C protein significantly hampered the effect of exogenous IFNs and promoted the replication of PPRV, EMCV, and VSV as indicated in Fig. 5, panels a, b, and c, respectively which highlights the crucial role of the C protein in causing infection and immune evasion. The results of the current study are congruent with previous studies on the C proteins of other viruses. Studies indicated that Morbillivirus RPV C protein inhibits the activation of the IFN-β promoter, and blocking C protein in the recombinant RPV led to much greater expression of IFN-β promoter [43]. A similar mechanism of Morbillivirus C protein in a recombinant MV, lacking C expression has previously indicated an increase in IFNs induction [28]. Studies on MV or RPV with knockout C, which rendered reduced growth in interferon-producing cells than V knockout equivalent viruses were also been reported previously [26, 48, 49].

Furthermore, this study finds that PPRV C protein can also inhibit the phosphorylation of STAT1, as revealed by three independent experiments (Fig. 6) and thereby hinders the signal transmission in JAK-STAT signaling pathway and impairs the host antiviral responses.

**Fig. 7:**
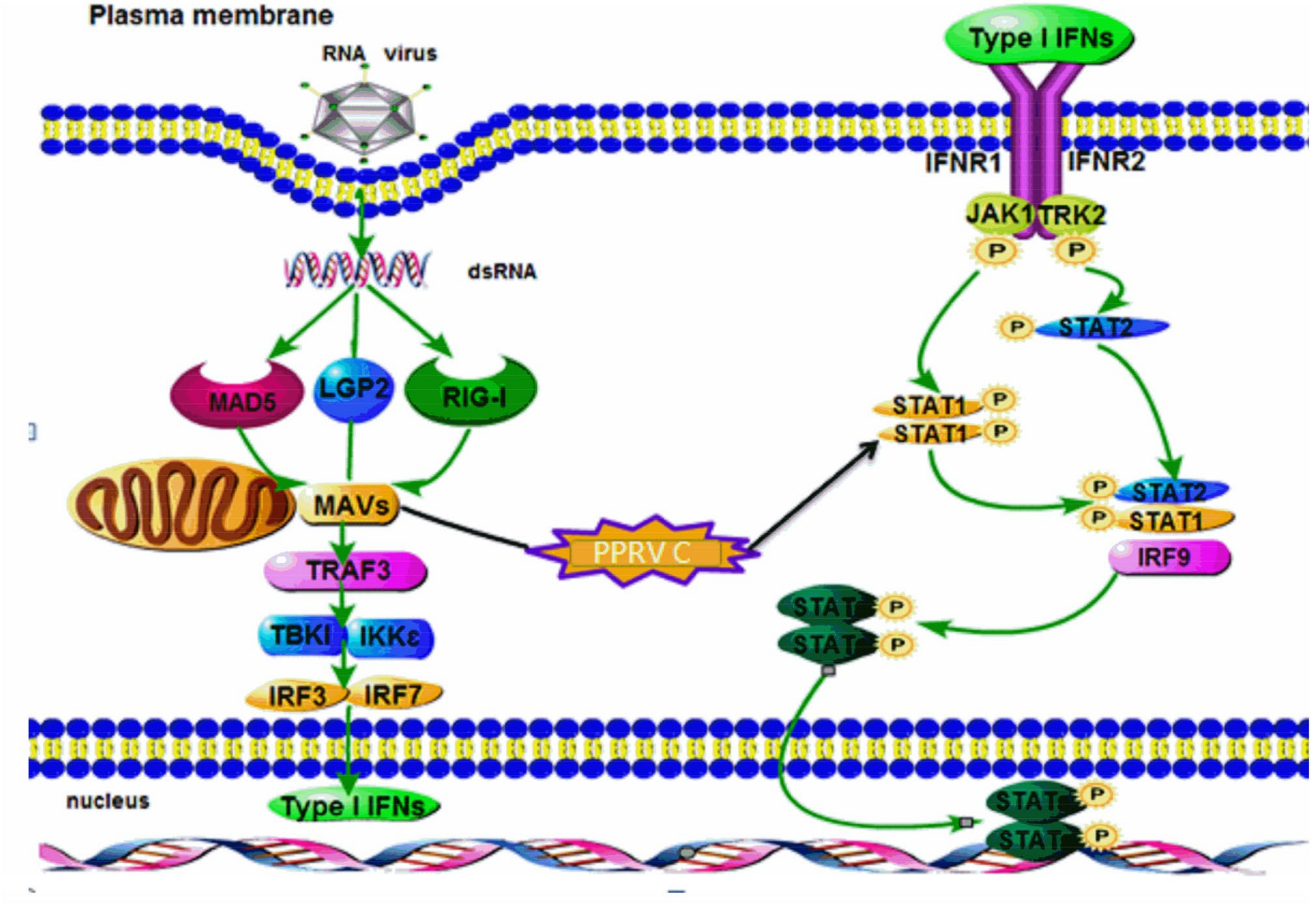
Mechanistic illustration of the interaction of PPRV C protein. General mechanism of PPRV entry, activation of various pathways, MAVS, JAK-STAT and IFN-β induction among others have been shown

In summary, this study communicates a comprehensive report on the role of the C protein of PPRV in immune evasion and diseases progression via offsetting endogenous and exogenous IFN responses by potentially interacting with RIG-1 and MAVS and by inhibiting the phosphorylation of STAT1 which disrupts the JAK-STAT signaling pathway in vitro (Fig. 7). Our findings emphasize on the C protein as a potential vaccine and therapeutic target and warrant further study in the same domain.

## Data Availability

The original data supporting the conclusions of this manuscript will be provided by the authors to any qualified researcher without reservation.
